# The Seasonality and Spatial Landscape of the Historical Climate-Based Suitability of *Aedes*-Borne Viruses in Four Atlantic Archipelagos

**DOI:** 10.3390/v17060799

**Published:** 2025-05-30

**Authors:** Martim A. Geraldes, Marta Giovanetti, Mónica V. Cunha, José Lourenço

**Affiliations:** 1 Católica Biomedical Research Center, Católica Medical School, Universidade Católica Portuguesa, 2780-156 Oeiras, Portugal; 2 Sciences and Technologies for Sustainable Development and One Health, Università Campus Bio-Medico di Roma, Via Álvaro del Portillo 21, 00128 Rome, Italy; 3 Oswaldo Cruz Institute, Oswaldo Cruz Foundation, Rio de Janeiro 21040-900, Brazil; 4 Climate Amplified Diseases and Epidemics (CLIMADE) Americas, Brazil; 5 Centre for Ecology, Evolution and Environmental Changes (cE3c) & CHANGE—Global Change and Sustainability Institute, Faculdade de Ciências, Universidade de Lisboa, 1749-016 Lisboa, Portugal; 6 Biosystems & Integrative Sciences Institute (BioISI), Faculdade de Ciências, Universidade de Lisboa, 1749-016 Lisboa, Portugal; 7 Climate Amplified Diseases and Epidemics (CLIMADE) Europe, Portugal

**Keywords:** dengue virus, epidemiology, suitability modelling, climate, *Aedes*-borne

## Abstract

While archipelagos have a demonstrated role in the stepping-stone process of the global dissemination of *Aedes*-borne viruses, they are often neglected in epidemiological and modelling studies. Over the past 20 years, some Atlantic archipelagos have witnessed a series of *Aedes*-borne viral outbreaks, prompting inquiries into the local historical suitability for transmission. In this study, the climate-based suitability for transmission of *Aedes*-borne viruses between 1980 and 2019 across Madeira, the Canaries, Cape Verde, and São Tomé e Príncipe archipelagos was estimated. For each island, we characterized the seasonality of climate-based suitability, mapped the spatial landscape of suitability, and quantified the historical effects of climate change. Results show that both island-level suitability and the historical impact of climate change decrease with distance from the equator, while significant seasonality patterns are observed only in subtropical climates. This study provides a unique historical perspective on the role of climate in shaping *Aedes*-borne virus transmission potential in Atlantic archipelagos. The findings herein described can inform local public health initiatives, including human-based prevention, targeted viral surveillance, and mosquito control programs.

## 1. Introduction

*Aedes*-borne viruses like Dengue, Zika, and Chikungunya have impacted communities and strained healthcare systems in numerous countries, disproportionately harming the most vulnerable populations. Among these, the Dengue virus (DENV) is the etiological agent of the most prevalent viral disease spread by *Aedes* mosquitoes and is believed to have originated in Southeast Asia [1,2]. Over 3.9 billion individuals across more than 132 nations are at risk of developing dengue, with an estimated 100–400 million infections, around 96 million symptomatic cases, and approximately 40,000 fatalities globally each year [3]. Additionally, there has been a significant rise in the global population residing in highly suitable regions for DENV transmission [4]. In parallel, the Zika virus (ZIKV) was first reported in Uganda in 1947 [5] and remained a neglected *orthoflavivirus* until 2007 due to its mild symptoms and limited prevalence in Africa and Asia [6]. Between 2013 and 2014, it was introduced into Brazil and other regions of the Americas [7], rapidly spreading across the globe, causing millions of cases, along with localized epidemics of fetal microcephaly and other congenital anomalies [6,8]. The Chikungunya virus (CHIKV) has also expanded geographically over the past 15–20 years [9]. It was first isolated during an outbreak in southern Tanzania in 1952–53 [10]. In 2004, it spread throughout the tropics and subtropics, affecting millions of people and becoming a global public health issue [11].

Africa, believed to be the origin of both ZIKV and CHIKV, is also indigenous to more than 30 other mosquito-borne viruses [12]. Historically, however, records suggest a limited number of arboviral diseases in Africa [13]. Since the 2000s, the prevalence of these diseases surged, likely due to a combination of urbanization, the spread of invasive *Aedes* mosquitoes, and heightened awareness [14], as exemplified by the major DENV and CHIKV outbreaks in 2007 in Gabon [15], the 2013 DENV Angola outbreak [15], the 2016 DENV outbreak in Burkina Faso [16], and the 2019 CHIKV epidemic in the Democratic Republic of Congo [11]. Furthermore, a 2020 study in Cameroon suggested that DENV prevalence in Africa may be higher than expected based on the currently limited surveillance [17]. Climate change projections indicate that Africa may become the continent most affected by rising temperatures [18], and modelling suggests that DENV activity may increase in the Global South, particularly in Asia and West Africa [4]. It has also been argued that, in Africa, environmentally driven changes (e.g., warming temperatures) could shift the disease burden from malaria to *Aedes*-borne arboviruses [18,19].

Archipelagos are known to play a pivotal role in the intercontinental spread of *Aedes*-borne viruses (e.g., ZIKV crossed the Pacific, island by island, before reaching the Americas [20]). At the same time, archipelagos often consist of relatively small geographic areas with diverse microclimates, making them promising settings to explore climate effects on local *Aedes*-borne virus suitability. Even so, archipelagos are not frequently the focus of modelling suitability studies, and they are often excluded from large spatial scale studies covering continents or the globe. In this study, we sought to characterize how historical climate conditions have shaped the suitability for *Aedes*-borne transmission in four Atlantic archipelagos: Madeira, the Canaries (Canary Islands), Cape Verde, and São Tomé e Príncipe.

Madeira is a North Atlantic Ocean archipelago composed of one main island with the same name, a smaller island called Porto Santo, and several inhabited islets. It is known for its mountainous landscape and subtropical climate, locally referred to as the eternal spring. All four seasons are present, and rainfall is more abundant during the winter. In 2005, *Aedes aegypti* became established in Madeira [21]. By 2012 it was widespread to the entire southern coast, and, in the same year, it was responsible for the first sustained European DENV outbreak. The total cases surpassed 2000, with higher incidence along the southern coast [22]. The virus was identified as DENV serotype 1 (DENV-1) and was likely imported from Venezuela via travelers [23]. As of today, the mosquito population remains endemic on the island [24].

São Tomé e Príncipe is a small equatorial archipelago in the Gulf of Guinea. It comprises two main islands, São Tomé (the most populated) and Príncipe. Temperature and humidity are high year-round, making it a tropical climate, with rainy and dry seasons. The rainy season goes from September to May, with rainfall peaks in October to November (major peak) and April to May (minor peak) [25]. In 2016, a serological survey found a 40% DENV seropositivity among pregnant women, indicating viral circulation before 2003–2004, when the serum samples were collected [26]. In 2022, São Tomé reported its first DENV outbreak, caused by DENV serotype 3 (DENV-3), leading to almost 1200 reported cases over 35 weeks. Phylogenomic analysis identified the genotype III American-II lineage, likely introduced from the Americas, supported by the index patient’s recent travel to Guadeloupe [27]. *Aedes aegypti* and *Aedes albopictus*—both primary vectors for DENV, ZIKV, and CHIKV—have been detected in all districts. *Aedes aegypti* is particularly abundant in Água Grande, the most urbanized district of São Tomé e Príncipe [28]. However, recent data suggest a notable increase in the proportional representation of *Aedes albopictus* compared to *Aedes aegypti* over time [29].

The Canary Islands archipelago is a group of seven islands along the northwestern coast of Africa. They are marked by a warm subtropical climate with varying levels of aridity that increase with proximity to the African coast, going from hot semi-desert to hot desert climate. A mild and cooler season and a hot and dry season can be depicted. In 2017, *Aedes aegypti* was detected in Fuerteventura and was eradicated in 2019 after a fast intervention from the local authorities [30]. In 2024, *Aedes aegypti* was recorded as newly introduced in Gran Canaria, and *Aedes albopictus* was first introduced in Tenerife [24,31]. Officially, there are no confirmed cases of locally acquired *Aedes*-borne diseases. Nevertheless, *Aedes aegypti* was considered established in the archipelago up to 1960, and an outbreak back in 1865 was attributed to DENV [32].

Cape Verde is a Central Atlantic ocean archipelago southwest of the Canary Islands. It is composed of several islands divided into two groups (windward and leeward) based on their wind exposure. On average, the climate is dry tropical, marked by warm temperatures and low rainfall. There are two seasons; the dry season from November to July, and the rainy season from August to October. The first reported DENV outbreak occurred in 2009, involving DENV-3 and DENV-4 serotypes that likely originated from West Africa [33,34]. The first ZIKV epidemic with associated microcephaly cases in Africa was reported in Cape Verde between 2015 and 2016, likely imported from circulating strains in the Americas [35]. A vector competence study conducted in the island of Santiago following the ZIKV outbreak revealed that the local *Aedes aegypti* population held high vector competence for DENV-2 and DENV-3 strains and low susceptibility to DENV-1 and DENV-4 [36]. It was also discovered that there was a high DENV infection rate in Santiago’s *Aedes aegypti* population, as well as serotype co-circulation, but a low level of autochthonous cases [37]. In 2023, DENV-3 reemerged, with phylogenetic relationships to an Asian clade unlike those from West Africa [38].

It is known that mosquito- and arboviral-trait dynamics are strongly influenced by real-time variation in local climatic variables such as temperature, humidity, and rainfall. The accumulation of empirical data from laboratory-controlled experiments on mosquitoes (e.g., *Aedes aegypti*) and viruses (e.g., dengue) in recent years has allowed the derivation of mosquito-viral mathematical expressions that adequately capture such non-linear responses to climatic variables (e.g., see [39,40]). For example, temperature exhibits an inverted, u-shaped effect on *Aedes aegypti* physiological and behavioral traits, with optimal ranges between 27 and 31 °C, maximizing biting rates, viral incubation efficiency, and survival, while extremes below 15–20 °C or above 35 °C sharply reduce fitness associated with such traits [39,40]. Similarly, while moderate to high levels of relative humidity enhance adult survival, egg production, and oviposition activity, desiccation at low humidity or physiological stress at high humidity impair these traits [41,42]. Such knowledge has proven key for modelling approaches aiming at estimating transmission potential and/or epidemic risk in both endemic and non-endemic regions of the globe (e.g., [43,44]).

To explore climate-based suitability for *Aedes*-borne virus transmission in these archipelagos, in this work, we used a mechanistic suitability measure known as index *p* [45]. *p* is informed by temperature and relative humidity and has been shown to effectively represent the spatio-temporal transmission dynamics of DENV [4,46,47], ZIKV [45,48] and CHIKV [45,49]. Under a series of analyses focusing on either the temporal or spatial scales, we examined the historical landscape of climate-based transmission suitability from 1980 to 2019. For each island of each archipelago, we characterized seasonal trends, mapped suitability spatially, and quantified the influence of historical climate change. The identified time windows and spatial hotspots of heightened transmission retrieved in this work can potentially inform local public health initiatives, such as human-based prevention, targeted viral surveillance, and mosquito control programs.

## 2. Materials and Methods

### 2.1. Aedes-Borne Viral Outbreak Data

To gather historical data regarding *Aedes*-borne epidemics in the Atlantic archipelagos, a review of the literature and health reports from national health authorities (e.g., official websites) was conducted. PubMed was queried for articles related to DENV, CHIKV, or ZIKV in São Tomé e Príncipe, Cape Verde, Madeira, and the Canaries using the query “(“Dengue” OR “Zika” OR “Chikungunya”) AND (“Cape Verde” OR “Cabo Verde” OR “São Tomé” OR “São Tomé e Príncipe” OR “Madeira” OR “Canaries” OR “Canary Islands”)” in all fields. Outbreak data were extracted from: reliefWeb [50] for DENV in Cape Verde 2009; the research paper [22] for DENV in Madeira 2012; the research paper [35] for ZIKV in Cape Verde 2015–2016; national bulletins [51] for DENV in São Tomé e Príncipe 2022 (in Portuguese); and national bulletins [52] for DENV in Cape Verde 2024–2025 (in Portuguese).

### 2.2. Transmission Suitability, Altitude, and Climate Data

To estimate the climate-based suitability for *Aedes*-borne virus transmission, a previously developed mosquito-borne viral suitability measure was used, referred to as index *p*. This measure’s theory and practice have been previously described in full by Obolski et al. [45] and Nakase et al. [4,46]. It can be interpreted as a proxy for the transmission potential of an individual adult female mosquito provided that susceptible hosts, the virus, and its vectors are present. It has been effectively utilized to describe the transmission potential and epidemiology of West Nile virus [53,54,55,56,57], CHIKV and ZIKV [47,58], and DENV [45,48,58,59]. Formally, the index *p* considers a combination of mosquito and viral traits that are known to be dependent on temperature, humidity, or both, including biting rate, mosquito-human and human-mosquito transmission probability per bite, human and mosquito incubation periods, human infectious period, and mosquito and human lifespans. Its mathematical formulation follows the classic basic reproduction number (R0) mathematical expression under a susceptible, incubating, infectious, and recovered (SEIR) transmission framework with an explicit adult mosquito population. Biological priors for particular traits (e.g., mosquito lifespan, incubation period, and biting rates), as well as the mathematical formulations on how the aforementioned traits vary in real time with the climatic variables, are described in detail in the study by Nakase et al. [40].

In this study, the same approach as in Nakase et al. (2023) was used by applying the same quantitative epidemiological priors (parameter values) [46]. While the climate-based suitability for *Aedes*-borne viruses is explored in this study, the index *p* is calibrated with biological priors specific to DENV, as performed by Nakase and colleagues [4,46]. Differences between DENV, ZIKV, and CHIKV are expected, but these are anticipated as minimal due to the similar range of the biological priors (e.g., incubation period, infectious period, human lifespan, etc.).

To calculate index *p*, climate data were downloaded from CHELSA v2.1 at high resolution at www.chelsa-climate.org. Data for temperature, humidity, and rainfall had a time resolution of 1 month (1980–2019) at a high spatial resolution of 30″ × 30″ [60]. As suggested by Nakase et al.’s previous work [4,46], we here assumed an index *p* > 0.5 as a proxy for high climate-based suitability. For the archipelago of Madeira, which has two inhabited islands (Madeira, Porto Santo), we analyze solely the main island of Madeira due to insufficient climatic data for Porto Santo. For Cape Verde, the island of Santa Luzia is analyzed together with the island of São Vincente due to its small size and the fact that it belongs to the same municipality (legislative border) of São Vincente. Small islets were not analyzed due to low resolution in climatic data but are mapped in figures for completeness.

Altitude data (elevation) featured in some of the analyses was obtained from WorldClim v2.1 at www.worldclim.org (accessed on January 2025).

### 2.3. Transmission Suitability Long-Term Trends

For estimating the long-term climate suitability trends, the seasonal Mann–Kendall (MK) test of Nakase et al. (2024) was applied to the transmission suitability index time series [4]. The MK test is a non-parametric method to identify monotonic trends in data, while the season variation incorporates seasonality considerations. To calculate the change rate for each pixel, Sen’s slope was utilized, offering a reliable assessment of linear change over time [61]. To address time series autocorrelation effects, the prewhitening algorithm by Yue et al. was applied [62]. This method involved detrending the time series, eliminating lag-1 autocorrelation, and subsequently reintegrating the trend to create prewhitened data. Following this adjustment, the seasonal MK test was conducted on the revised time series, with significance evaluated at a false discovery rate of less than 0.10 for all geo-located pixels (latitude–longitude locations).

## 3. Results

### 3.1. Aedes-Borne Outbreak Dynamics and Climate-Based Suitability Across the Archipelagos

**Figure 1:**
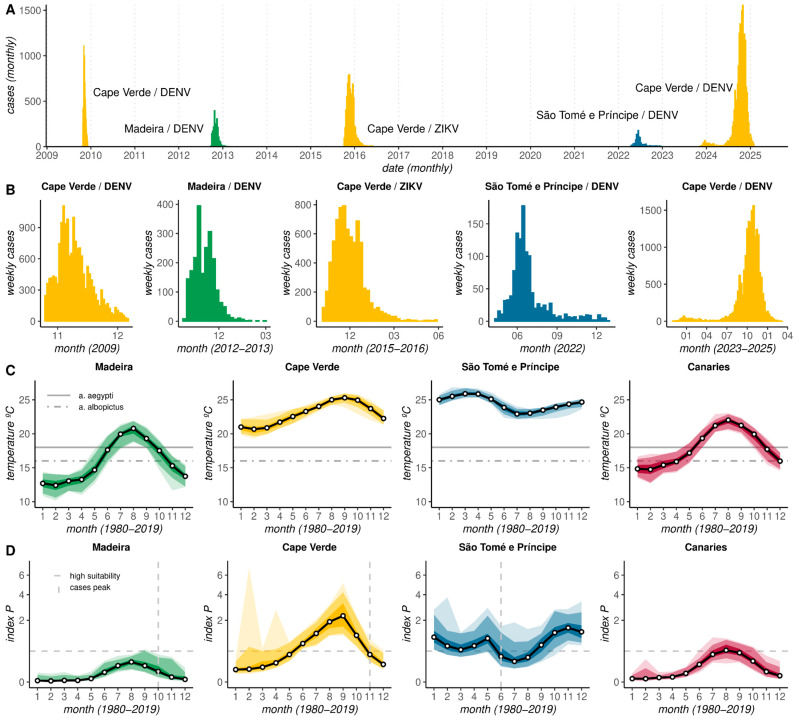
Temporal dynamics of *Aedes*-Borne virus transmission and climate-based suitability in Atlantic Archipelagos. (**A**) A timeline depicting documented outbreaks of *Aedes*-borne viruses (DENV, ZIKV) on the islands of Madeira, the Canaries, Cape Verde, and São Tomé e Príncipe. (**B**) Weekly outbreak curves per unique combination of archipelago and virus. (**C**) Distributions of historical monthly temperatures, with horizontal lines indicating the lower end of adequate temperature ranges for CHIKV, ZIKV, and DENV transmission by *Aedes aegypti* (full) and *Aedes albopictus* (dashed). (**D**) Distributions of historical monthly climate-based suitability (index *p*), with horizontal line marking the threshold *p* = 0.5 as proxy for high suitability and vertical lines marking the months for which peak outbreak sizes were reported at each of the archipelagos. (**C**,**D**) Shaded color areas represent, in increasingly darker shades, the min/max range, 95% and 50% percentiles.

Since the early 2010s, a few *Aedes*-borne viral outbreaks have taken place in the Atlantic archipelagos (Figure 1A,B). The island of Madeira experienced the first European locally transmitted DENV outbreak in 2012. Cape Verde had a DENV outbreak in 2009, a ZIKV outbreak in 2015, and a long-lasting, large DENV outbreak between 2023 and 2025. São Tomé and Príncipe reported its first DENV epidemic in 2022. The Canaries have not experienced an outbreak yet, and CHIKV remains unreported in all archipelagos.

Ranges of adequate and optimal temperature levels have been unraveled in a number of previous studies [63]. Of particular interest are the range and optimal temperature for transmission of DENV, ZIKV, and CHIKV by *Aedes aegypti* and *Aedes albopictus*. Mordecai and colleagues have reported a range of 18–35 °C for *Aedes aegypti* and 16–32 °C for *Aedes albopictus*, with optimal temperatures at 29 °C and 26 °C, respectively [39]. On the island of Madeira and across the Canaries, holding subtropical climates, historical temperatures (1980–2019) often breach the lower level of the adequate temperature range for both mosquito species in the summer months (Figure 1C). Given the slight differences in adequate temperature ranges between the mosquito species, the seasonal window adequate for transmission is wider for *Aedes albopictus* than *Aedes aegypti* in both archipelagos. In contrast, in tropical Cape Verde and São Tomé e Príncipe, historical temperatures remained above both the lower and optimal thresholds year-round (Figure 1C). A possible implication of these observations is that endemicity could be supported in the tropical archipelagos, while only epidemic behavior should be expected in the subtropical archipelagos.

Using index *p*, a mechanistic measure of climate-based mosquito-borne viral suitability used as a proxy for transmission potential (see Section 2), the historical monthly suitability for each island (Supplementary Figure S1) and archipelago (Figure 1D) was estimated. Summarizing time series as monthly distributions provided a means to explore the typical seasonal signal. The subtropical archipelagos (Madeira, Canaries) presented much lower suitability throughout the year compared to the tropical archipelagos (Cape Verde, São Tomé e Príncipe). In fact, the threshold of *p* = 0.5, a proxy for high suitability [46], was never breached in Madeira, barely breached in the Canaries between July and September, and breached throughout the year in Cape Verde and São Tomé e Príncipe. There were also clear seasonal trends, with a typical single-peak signal in Madeira, Cape Verde, and the Canaries, with maximums observed during months related to the summer season in the northern hemisphere (June-October). For São Tomé e Príncipe, an atypical, weak, double-peak seasonal trend was unraveled, including a short-lasting peak in May and long-lasting peak between October and January.

Considering the few reported outbreaks to date (Figure 1A), a time lag was observed between the peaks of climate-based suitability and reported cases (Figure 1D). On the island of Madeira, suitability peaked 2 months before the 2012 DENV outbreak. In Cape Verde, this lag was 1 month for DENV and 2 months for ZIKV. In São Tomé e Príncipe, the lag was 1 month relative to the short-lasting suitability peak of May. The observation that climate-based suitability increases before reported cases has been well described elsewhere [46] and is an expected phenomenon related to the stacking of inherent individual- and population-level delays in responding to suitability (e.g., mosquito life-cycle, transmission generation time, symptoms, individual testing, etc).

### 3.2. Geographic Variation in Climate-Based Suitability Across the Archipelagos

**Figure 2:**
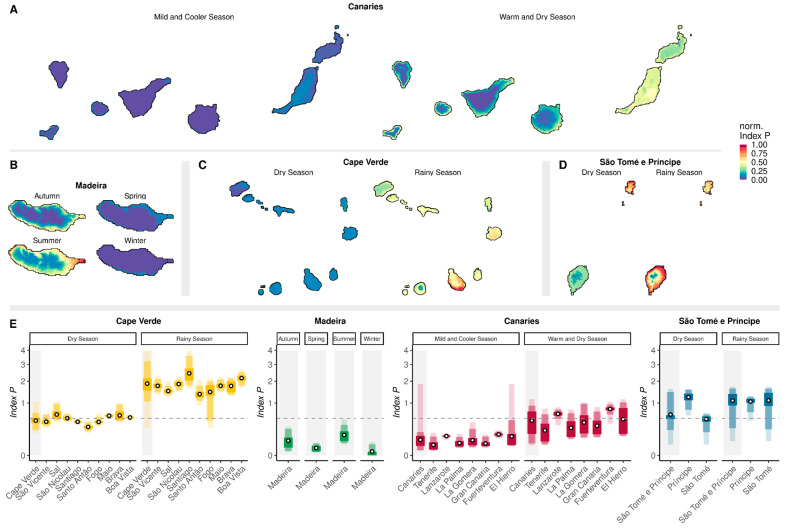
Historical spatial and seasonal distributions of estimated climate-based suitability (1980–2019). (**A**–**D**) Maps showing the spatial distribution of mean historical climate-based suitability for each island of each archipelago during different local typical seasons: (**A**) Canary Islands, (**B**) Madeira, (**C**) Cape Verde, and (**D**) São Tomé e Príncipe. Suitability is normalized to the archipelago maximum for visualization. (**E**) Distributions of estimated historical climate-based suitability per island for each archipelago (gray background) and islands (white background), with medians represented by points and shaded color areas representing, in increasingly darker shades, the min/max range, 95% and 50% percentiles. The horizontal dashed line marks a high suitability threshold of *p* = 0.5.

Aggregating estimated historical climate-based suitability (1980–2019) into seasons (Supplementary Figures S2–S5) and months (Supplementary Figures S6–S9) revealed great heterogeneity both within and between archipelagos (Figure 2). As already reflected by the spatially aggregated results (Figure 1D), the highest suitability was estimated during the warm and dry season for the Canaries (Figure 2A), summer for Madeira (Figure 2B), and the rainy season for both Cape Verde (Figure 2C) and São Tomé e Príncipe (Figure 2D).

The islands of the subtropical archipelagos presented the lowest suitability ranges across all islands (Figure 2E). In Madeira, 0% of land was deemed highly suitable (*p* > 0.5) in all four seasons. In the Canaries, the proportion of land classified as highly suitable varied between nearly 0% (mild and cooler season) and ~44% (warm and dry season). In this season, the islands of Lanzarote and Fuerteventura showed the highest suitability and the largest proportion of land deemed highly suitable, at ~96% and >99%, respectively. During the same period, land deemed highly suitable reached 11.34% in Tenerife, ~14% in La Palma, ~16% in Gran Canaria, ~31% in La Gomera, and ~43% in El Hierro.

In Cape Verde, the island with the historically highest suitability was Santiago, where the capital is located, while the island with the lowest suitability was Santo Antão. During the rainy season, 100% of land across the islands was deemed highly suitable (*p* > 0.5), with the exception of Fogo island (~97%). In São Tomé e Príncipe, the island of Príncipe exhibited the highest historical suitability. Across seasons, the proportion of land deemed highly suitable in São Tomé ranged from ~62% to ~91%, while in Príncipe it varied less, from ~97% to >99%.

During the seasons with the highest suitability, all four archipelagos presented a notable coastal effect, with lower suitability often associated with the interior regions of some islands (Figure 2A–D).

### 3.3. Long-Term Trends in Climate-Based Suitability Across the Archipelagos

**Figure 3:**
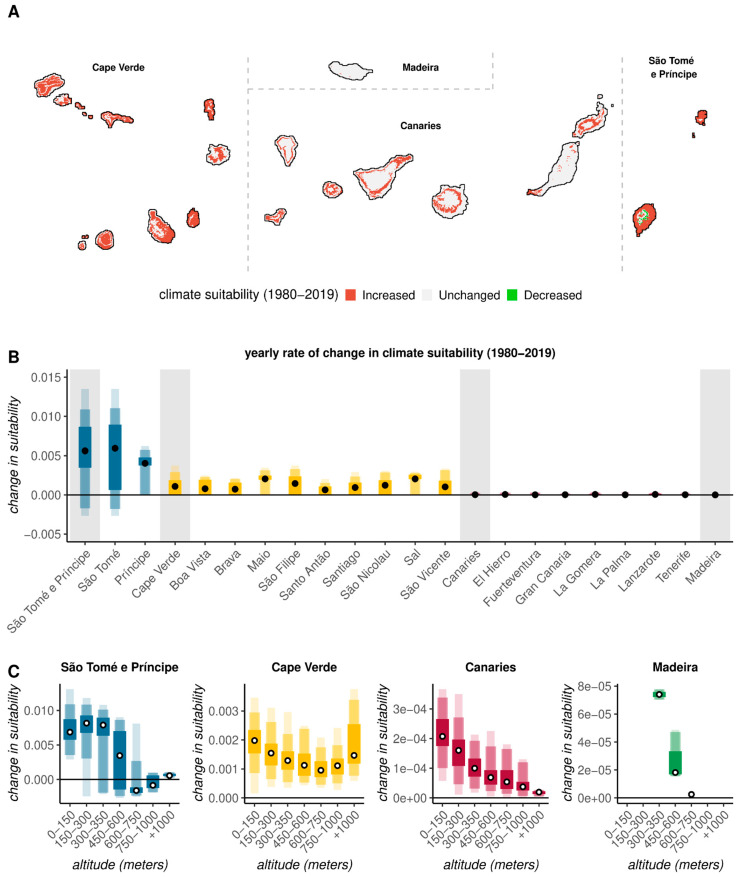
Historical trends in climate-based *Aedes*-borne suitability (1980–2019). (**A**) Mapping of climate suitability: increased (red), remained unchanged (gray), or decreased (green). Only locations (latitude–longitude) with statistically significant estimations of change are presented. (**B**) Distributions of estimated yearly changes (slopes) in suitability per archipelago (shaded gray areas) and islands. (**C**) Relationship between local altitude and estimated yearly changes (slopes) in suitability per archipelago. (**B**,**C**) Shaded color areas represent, in increasingly darker shades, the min/max range, 96% and 50% percentiles.

Resorting to the long-term time series of estimated historical climate-based suitability per location (latitude–longitude point), per island, and per archipelago, trends in suitability over the period 1980–2019 were quantified (see Section 2). Statistical significance was measured and mapped (Supplementary Figure S10), and the locations with statistically significant increasing or decreasing trends are presented in Figure 3A (for absolute rates of change, see Supplementary Figure S11).

All archipelagos showed evidence of historical increases in suitability due to long-term changes in climatic variables, but with significant differences within and between archipelagos in the proportion of land with suitability changes (Figure 3A). The proportion of land with historical suitability increases was ~79% in São Tomé e Príncipe, ~64% in Cape Verde, 24% in the Canaries, and <1% in Madeira archipelagos. Only São Tomé e Príncipe presented long-term decreases in climate-based suitability, amounting to ~6% of the territory on the island of São Tomé (Figure 3A,B).

When aggregated at the island level, all of the islands belonging to the tropical archipelagos (São Tomé e Príncipe, Cape Verde) showed significant long-term increases in climate-based suitability (Figure 3B). Increases in suitability were particularly noticeable in São Tomé e Príncipe, being higher on the island of São Tomé (~47% higher than in Príncipe). In Cape Verde, the islands of Maio, Sal, and São Filipe featured in the top three with the highest increases in suitability (Figure 3B). In contrast to the islands of the tropical archipelagos, all of the islands of the subtropical archipelagos (Madeira, Canaries) presented unmarked changes in climate-based suitability (Figure 3B). For Madeira, the Canaries, and São Tomé e Príncipe, the local level of increase in spatial suitability over the past 40 years was negatively related to altitude (Figure 3C). In Cape Verde, this relationship presented instead in a U-shape, with lower and higher altitudes presenting the highest historical increases in suitability.

## 4. Discussion

Since the beginning of the 21st century, Atlantic archipelagos such as Madeira, Cape Verde, and São Tomé e Príncipe have experienced viral *Aedes*-borne outbreaks (DENV, ZIKV). It remains unclear whether the historical absence of such outbreaks resulted from limited transmission potential or limited awareness with non-differential diagnosis and, consequently, underreporting. Climate is widely recognized as a key factor influencing the epidemic activity and geographical spread of *Aedes*-borne viruses due to its impact on mosquito population dynamics and the viral extrinsic incubation period. In this study, we estimated climate-based transmission suitability through the climate-based suitability measure index *p*. Through the temporal and spatial dimensions of estimated suitability, we characterized the historical and current potential for *Aedes*-borne viruses on Atlantic archipelagos based on the sole contribution of climate.

Monthly estimates from 1980 to 2019 allowed us to reconstruct the typical year in terms of transmission suitability by analyzing broad monthly metrics (e.g., percentiles) relative to the threshold index *p* > 0.5, which is typically used as a proxy for high suitability. Across the archipelagos, three seasonal patterns were revealed: high suitability with either weak (São Tomé e Príncipe) or strong (Cape Verde) seasonality, and low suitability with strong seasonality (Madeira, the Canaries). The two regimes of low and high suitability matched local climate regimes, with high suitability in the tropical regime and low suitability in the subtropical regime. As such, the estimates of climate-based suitability indicated that increasing distance from the equator may be linked to an overall reduction in transmission suitability.

Analyses of estimated suitability indicated that peak suitability is likely between June and October for the archipelagos with strong seasonality (Cape Verde, Madeira, Canaries), and around October—January for the archipelago with weak seasonality (São Tomé e Príncipe). These months-long time windows aligned with the local rainy season for Cape Verde and São Tomé e Príncipe, the summer-autumn season for Madeira, and the warm and dry season for the Canaries. It is thus suggested that local surveillance and mosquito control initiatives could focus on these yearly time windows, during which viral introduction events may be more likely to result in transmission chains and potentially develop into outbreaks.

Madeira and the Canaries presented several months of the year with zero suitability for transmission, reinforcing the consensus that while such islands hold potential for *Aedes*-borne viral epidemic activity, their local climate may be historically inadequate to support endemicity. Indeed, the large 2012 DENV outbreak in Madeira ended with viral extinction during the locally colder winter months. In contrast, Cape Verde and São Tomé e Príncipe had no months with zero suitability, supporting the view that tropical climates are amenable not just to *Aedes*-borne viral epidemic activity but also potentially to endemicity.

The exploration of climate-based suitability was expanded to include the spatial dimension, complementing insights gained from solely analyzing seasonality. There was clear variation in the spatial distribution of suitability within and between the islands. For example, a clear coastal effect was observed in estimated suitability for many of the islands, with higher suitability associated with coastal areas. Volcanic islands, such as those from the studied archipelagos, typically have low-altitude coastal areas and high inland altitudes, resulting in localized phenomena (e.g., orographic, rain shadowing, etc.) that can generate highly heterogeneous local climates. Parallel to this, altitude negatively affects the development and fitness of *Aedes* species, such that their presence is typically associated with low to mid-elevations.

The archipelago of São Tomé e Príncipe, specifically the island of São Tomé, presented the strongest coastal effects, with suitability being highest along the coast, particularly in the northeast, where the capital and most human settlements are located. In accordance, epidemiological data from 2022 have shown that 68% of reported DENV cases occurred in the most northeastern district of Água Grande [28]. In Cape Verde, during the rainy season, coastal effects were also observed, particularly for the islands of Fogo and Santiago. In Fogo, suitability was estimated to be lowest in the center, coinciding with the high-altitude Pico do Fogo volcano at its center. In Santiago, higher suitability was estimated in the southwestern coastal line, which coincides with lower rainfall due to rain shadow caused by the central volcanic mountain, the location of the capital city, terraced farming, and the lowest-altitude plains on the island. Santiago, and in particular its southwestern region, seems in fact to be a hub for mosquito-borne virus activity according to human seroprevalence data against ZIKV, DENV, YFV, and CHIKV [64]. The island was also highly affected by DENV in the 2009 outbreak, by ZIKV in 2015–2016, and again by DENV during the 2024–2025 outbreak [35,64,65]. Within the Canaries, the same coastal effect was notoriously present for the western group of islands (La Palma, El Hierro, La Gomera, Tenerife, and Gran Canaria), coinciding with the islands’ high altitude variation between the coast and their inland center. In contrast, the coastal effect was not found for Lanzarote and Fuerteventura, which broadly present low-altitude landscapes. In Madeira, for similar reasons related to local altitude landscapes, suitability was also higher along the coast, in particular on the east–west poles. The broad coastal pattern observed in this study for Madeira is compatible with the estimated suitability from another study based on a mix of climatic, human, and land use conditions on the island [66]. The 2012 DENV outbreak burden was concentrated in the coastal line to the south, where the capital city is located, rather than across the entirety of the coastal line of the island, although it is recognized that the outbreak was widely underreported [66].

Over the studied timeframe (1980–2019), estimated climate-based suitability increased but with high variation in the absolute change accumulated over the years across islands. The level of suitability increase over the years decreased with distance to the equator. More specifically, within an island-level perspective, islands of archipelagos with tropical climates had clear suitability increases over the 40-year period, while islands with subtropical climates had negligible or no increases at all. The archipelago of São Tomé e Príncipe had the highest suitability changes, experienced in both of its two islands but more strongly in São Tomé. The latter was the only island for which statistically significant negative trends in suitability were estimated, concentrating on locations at the inner part of the island. In contrast, suitability increases on the island were concentrated in locations around the coast. In Cape Verde, suitability increases were more generally dispersed across the land areas of the islands.

In general, the outputs of this study suggest that higher altitudes may be associated with smaller historical increases in suitability, likely reflecting that climate would have to change significantly in such high-altitude areas to counterbalance the historically inadequate climate of such locations. In parallel to climate-driven increases in suitability, urbanization and globalization (e.g., enhanced trade and travel) trends also contribute positively to the epidemiology and activity of *Aedes*-borne viruses, as exemplified by the introduction of DENV in São Tomé e Príncipe from Guadalupe [27] and the introduction of ZIKV in Cape Verde from the Americas [35]. These processes working in tandem in the future will continue to raise the risk of viral importation, as well as the likelihood of locally sustained viral transmission chains on islands. In islands with a subtropical climate, this will raise the odds of short-term epidemic activity, while in islands with a tropical climate, it will raise the odds of endemicity.

Globally, there is an urgent need for adaptive vector control strategies tailored to specific climatic and geographical contexts, both in regions on the brink of suitability thresholds and in regions that are already suitable. Altogether, public health initiatives, whether focused on human-based prevention, viral surveillance and differential diagnosis, or mosquito control, should build up on predictions and estimations from computational studies on timing and locations of adequate or heightened suitability, such as those proposed in this study.

## Data Availability

The data presented in this study, which include spatial rasters of the estimated suitability across the islands of the studied archipelagos, are available on request from the corresponding author. The data used as input for the modelling is available in public repositories and there are restrictions on republishing that prevent making them available with this article.

## Supplementary Materials

The following supporting information can be downloaded at: https://www.mdpi.com/article/10.3390/v17060799/s1.

## Abbreviations

The following abbreviations are used in this manuscript:

DENV	Dengue virus
CHIKV	Chikungunya virus
ZIKV	Zika virus
YFV	Yellow fever virus
