# Supplementary material for: The Seasonality and Spatial Landscape of the Historical Climate-Based Suitability of Aedes-Borne Viruses in Four Atlantic Archipelagos
Source: Viruses. 2025 May 30;17(6):799. doi: 10.3390/v17060799 (PMC12197518; doi:10.3390/v17060799)
Supplement: Supplementary file 1 [file viruses-17-00799-s001.zip › viruses-3628527-supplementary.pdf]

Article

The seasonality and spatial landscape of the historical climate-based suitability of Aedes-borne viruses in four Atlantic archipelagos

Martim Afonso Geraldes, Marta Giovanetti, Mónica V. Cunha, José Lourenço

Supplementary Figures

Supplementary Figure S1 – Per island monthly transmission suitability index (Index P) curves presenting seasonal trends in suitability over the period 1981-2019.

Supplementary Figure S2 – Mean index P for the Madeira island in the period 1981-2019, separated by local seasons.

Supplementary Figure S3 – Mean index P for the São Tomé e Príncipe archipelago in the period 1981-2019, separated by local seasons.

Supplementary Figure S4 – Mean index P for the Cape Verde archipelago in the period 1981-2019, separated by local seasons.

Supplementary Figure S5 – Mean index P for the Canaries archipelago in the period 1981-2019, separated by local seasons.

Supplementary Figure S6 – Mean monthly index P for the Island of Madeira in the period 1981-2019.

Supplementary Figure S7 – Mean monthly index P for the São Tomé e Príncipe archipelago in the period 1981-2019.

Supplementary Figure S8 – Mean monthly index P for the Cape Verde archipelago in the period 1981-2019.

Supplementary Figure S9 – Mean monthly index P for the Canaries archipelago in the period 1981-2019.

Supplementary Figures S10 – Long-term transmission suitability trend p-values.

Supplementary Figures S11 – Long-term transmission suitability trend absolute values.

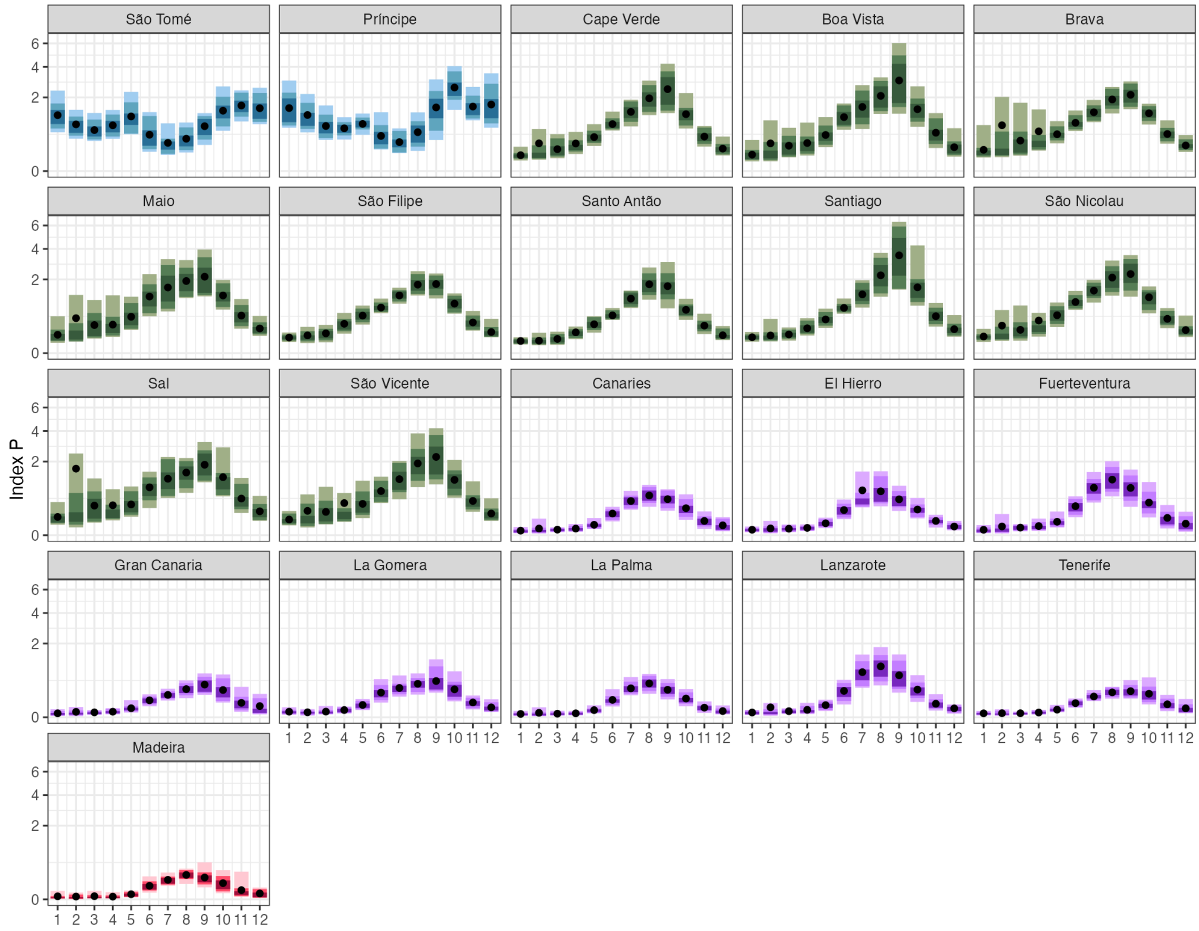

Supplementary Figure S1 – Per island monthly transmission suitability index (Index P) curves presenting seasonal trends in suitability over the period 1981-2019.

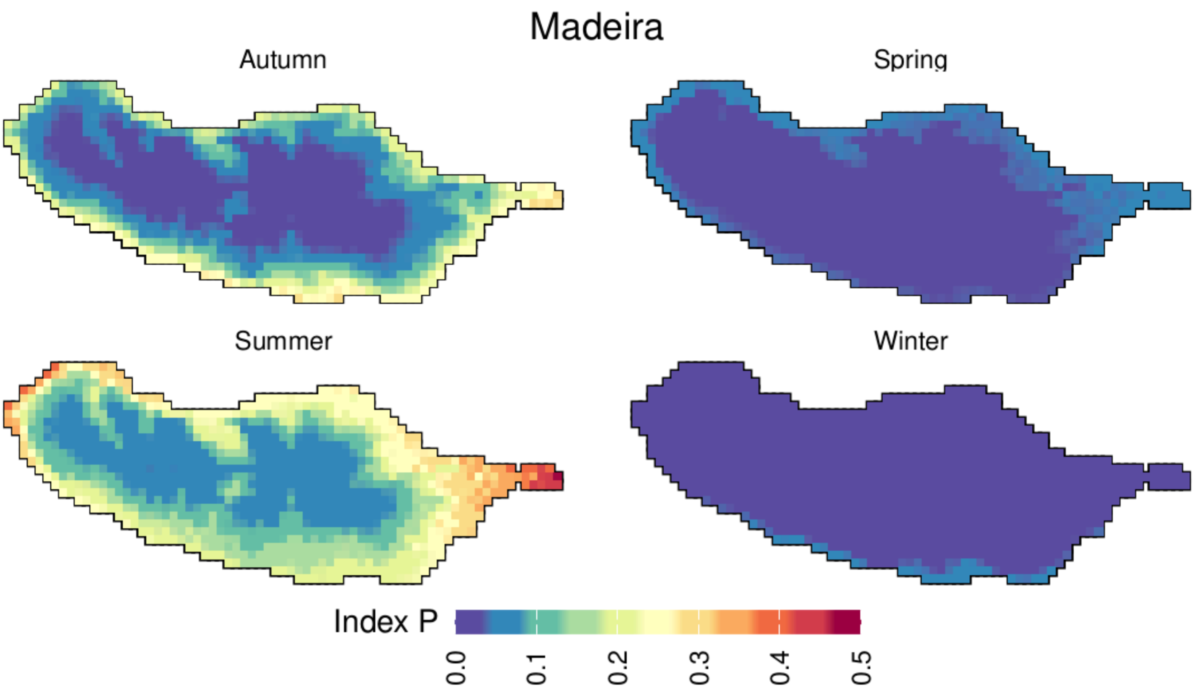

Supplementary Figure S2 – Mean index P for the Madeira island in the period 1981-2019, separated by local seasons.

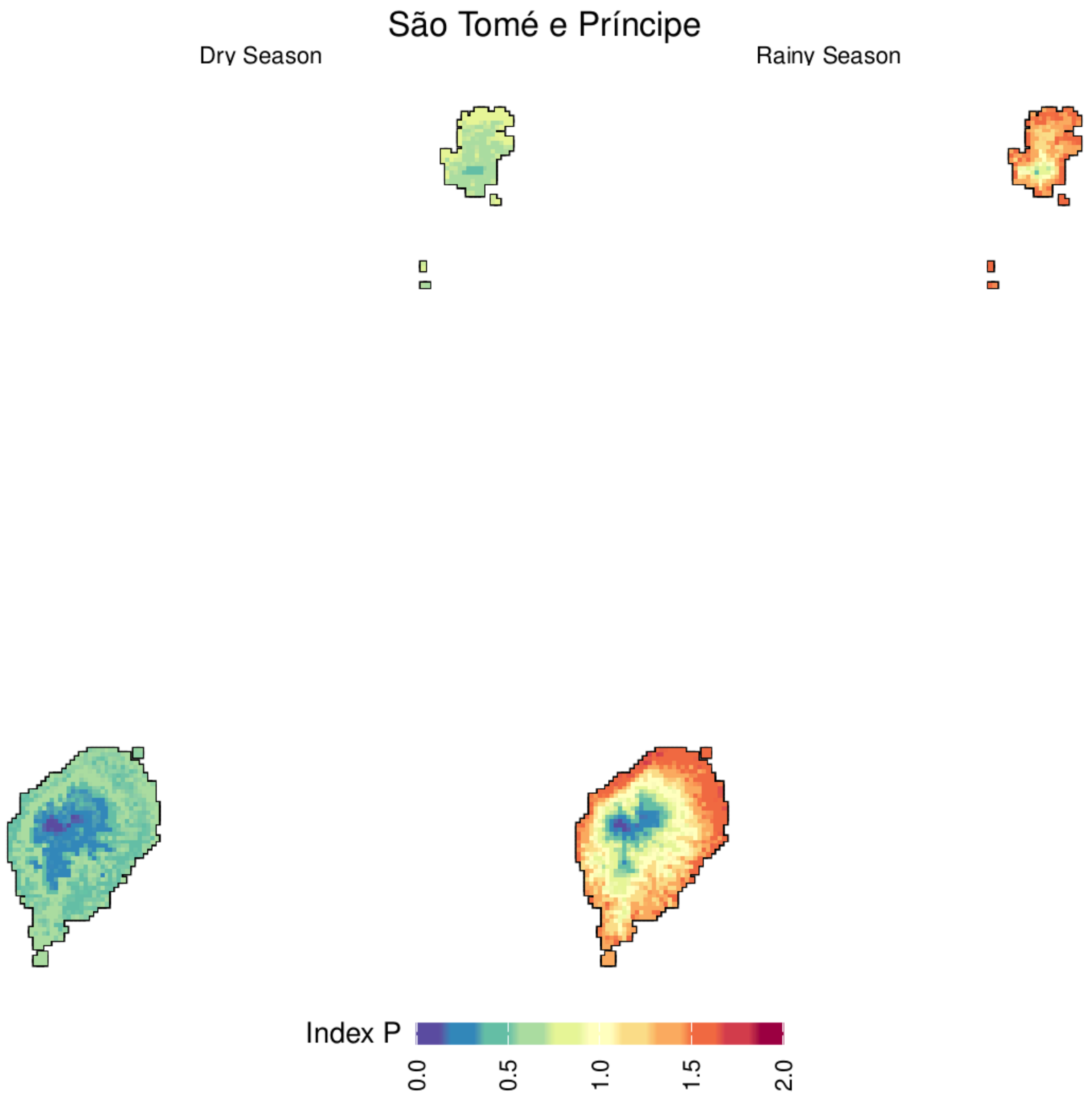

Supplementary Figure S3 – Mean index P for the São Tomé e Príncipe archipelago in the period 1981-2019, separated by local seasons.

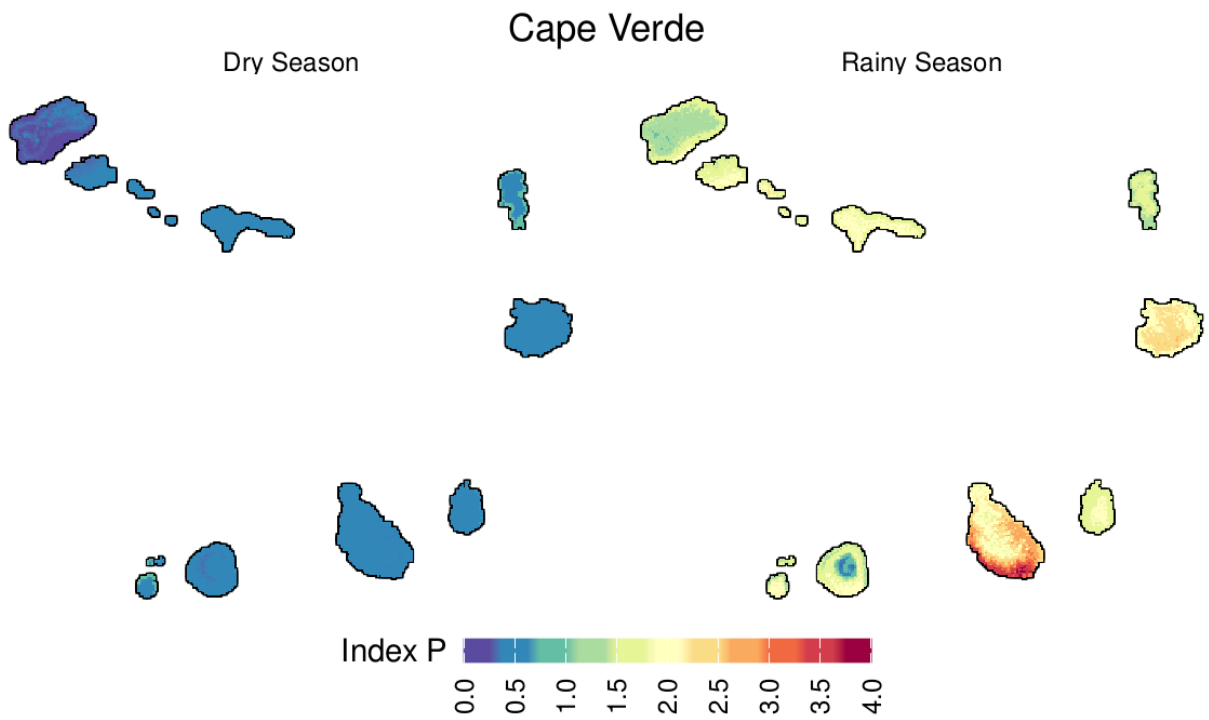

Supplementary Figure S4 – Mean index P for the Cape Verde archipelago in the period 1981-2019, separated by local seasons.

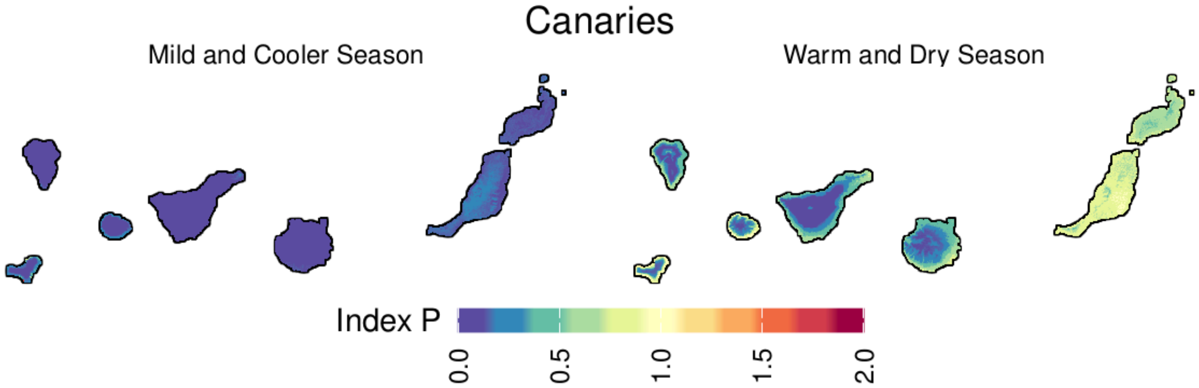

Supplementary Figure S5 – Mean index P for the Canaries archipelago in the period 1981-2019, separated by local seasons.

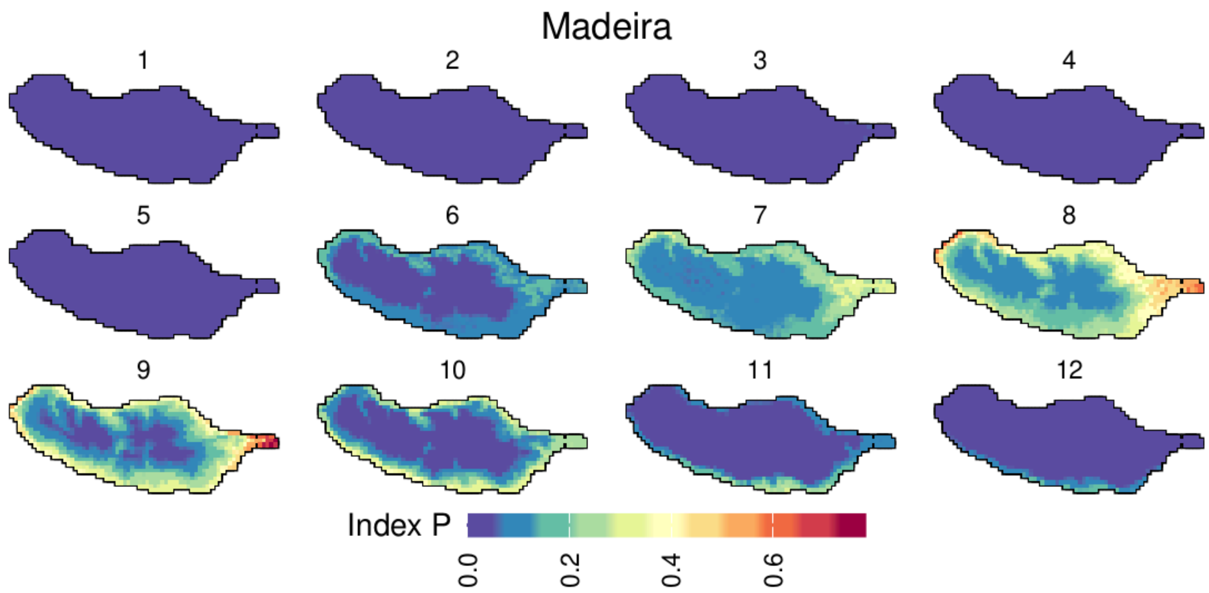

Supplementary Figure S6 – Mean monthly index P for the Island of Madeira in the period 1981-2019.

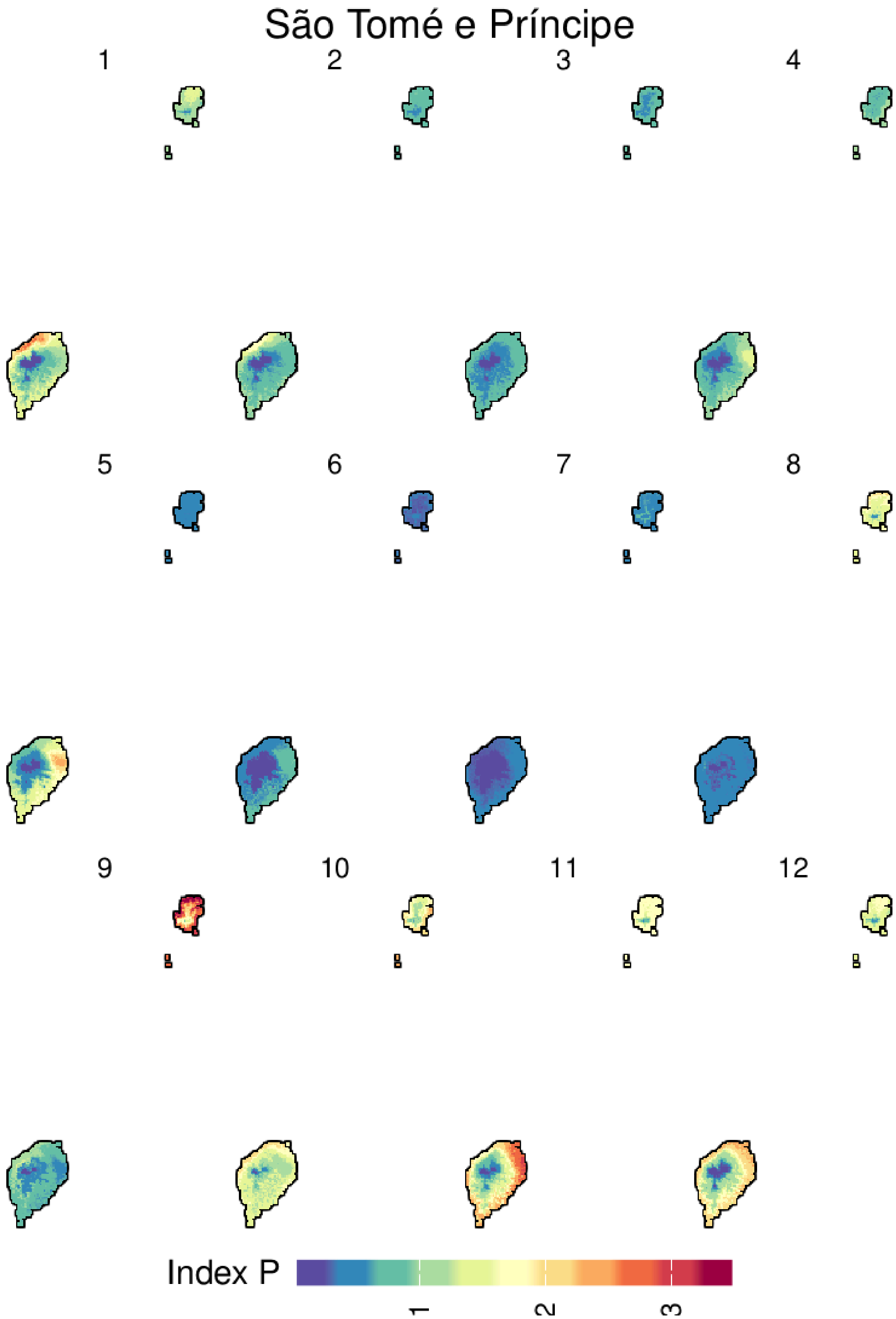

Supplementary Figure S7 – Mean monthly index P for the São Tomé e Príncipe archipelago in the period 1981-2019.

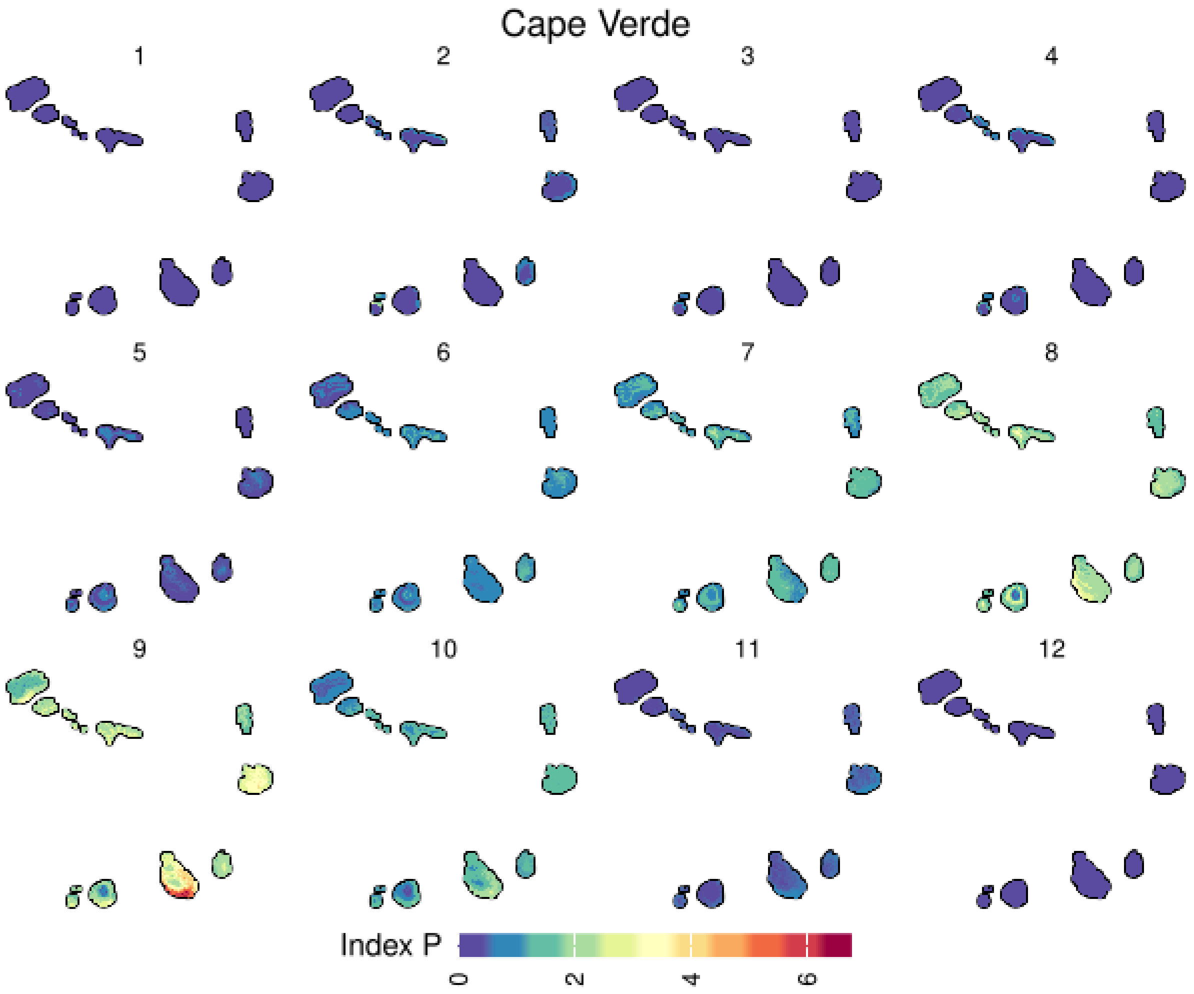

Supplementary Figure S8 – Mean monthly index P for the Cape Verde archipelago in the period 1981-2019.

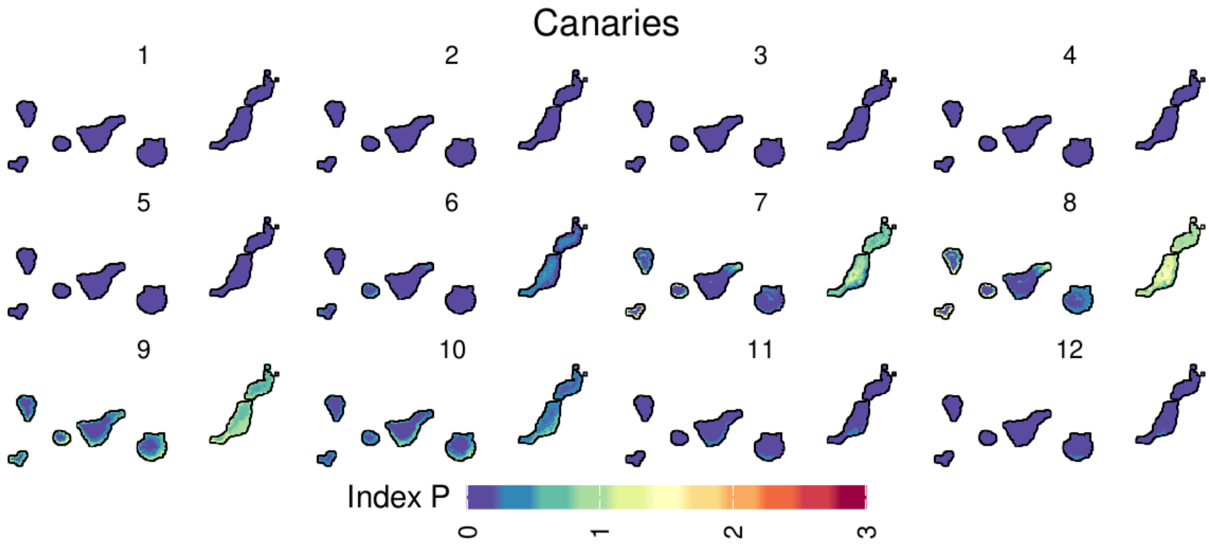

Supplementary Figure S9 – Mean monthly index P for the Canaries archipelago in the period 1981-2019.

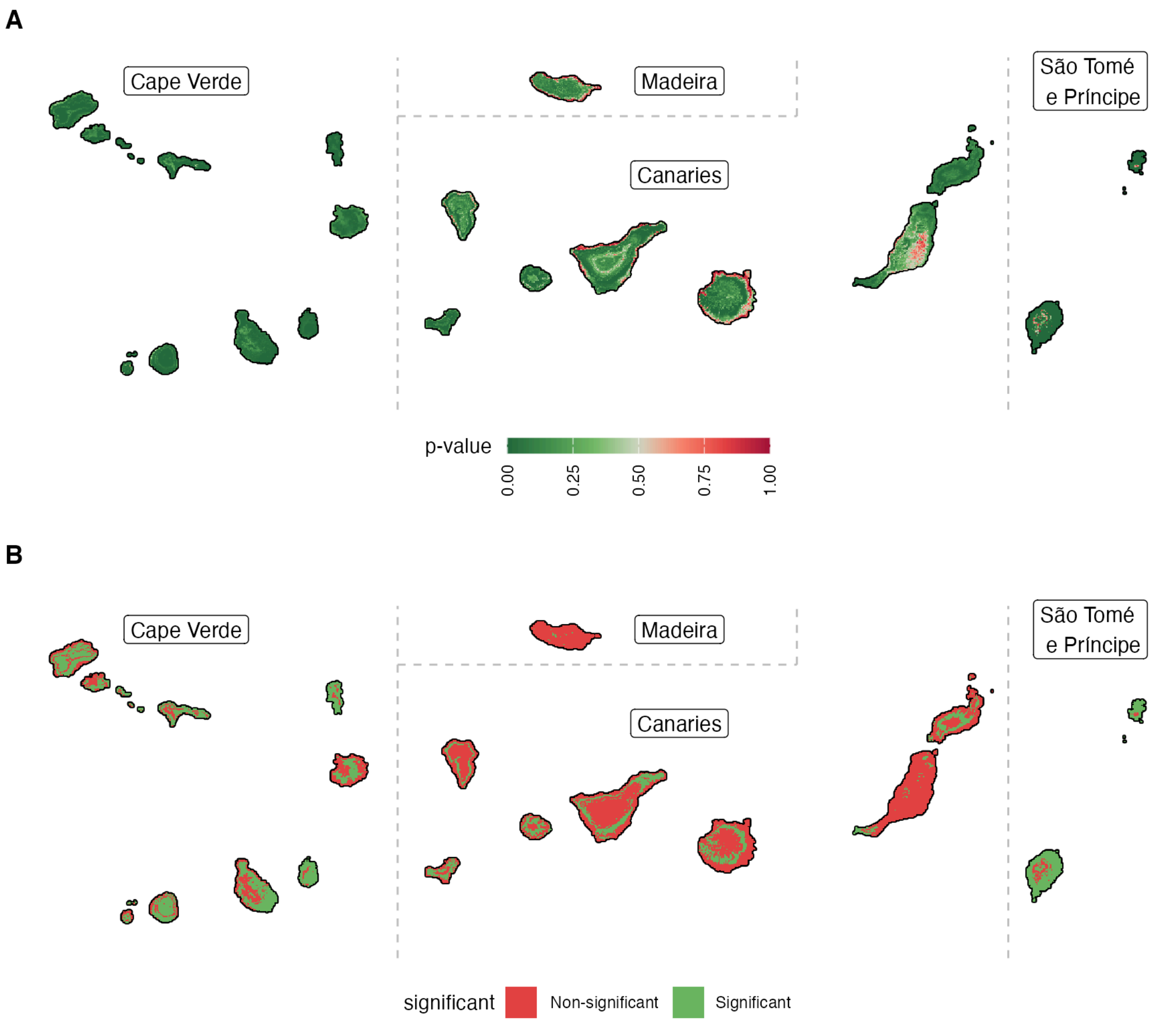

Supplementary Figures S10 – Long-term transmission suitability trend p-values.
(A) raw p-values per pixel; (B) Trend's significance status per pixel.

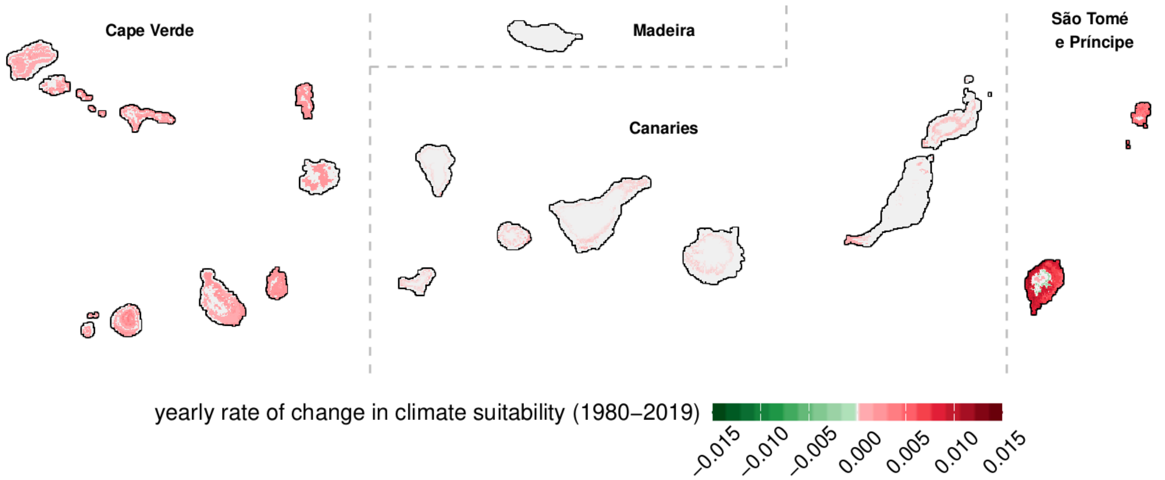

Supplementary Figures S11 – Long-term transmission suitability trend absolute values.
